# Gardenia Fruit as a Novel Substrate for Kombucha Fermentation: Impacts on Physicochemical Properties, Bioactive Compounds, Metabolomic Profiles, and Sensory Acceptance

**DOI:** 10.3390/foods15152670

**Published:** 2026-07-29

**Authors:** Kaiyu Chen, Qi Zhang, Yanming Ren, Qinxue Ni, Guangzhi Xu, Youzuo Zhang, Qiufen Mo

**Affiliations:** 1 College of Food and Health, Zhejiang A&F University, Hangzhou 311300, China; 2 National Key Laboratory for Development and Utilization of Forest Food Resources, Zhejiang A&F University, Hangzhou 311300, China; 3 Zhejiang Jiaozhi Technology Co., Ltd., Hangzhou 310021, China

**Keywords:** *gardenia* fruit, kombucha beverages, total polyphenols, untargeted metabolomics, sensory analysis

## Abstract

*Gardenia* fruit, a medicinal and edible resource rich in bioactive compounds, faces several limitations, including low extraction efficiency, poor stability, and intense bitterness. In this study, *gardenia* fruit powder at varying concentrations (2.5%, 5.0%, 7.5%, 10.0%, and 12.5%) was mixed with a kombucha inoculum and fermented for 8 days to develop functional beverages. The results demonstrated that fermentation with *gardenia* fruit powder effectively supported SCOBY growth. The 5.0% addition of *gardenia* fruit (G5Fer) produced thicker bacterial cellulose, higher total titratable acidity (10.43 g/L), and increased total polyphenols (37.12 mg GAE/100 mL) after 8 days of fermentation. G5Fer also achieved the highest sensory scores, especially in aroma, flavor, and overall acceptance. Higher doses of *gardenia* fruit (≥7.5%) caused turbidity, dark color, and strong sourness, leading to lower sensory scores. Non-targeted metabolomics revealed that kombucha fermentation reshaped the metabolic profile, enriching phenolic acids (e.g., gallic acid and caffeic acid) and flavonol glycosides (e.g., kaempferol-3-O-rutinoside) via the biosynthesis of phenylpropanoid pathways while antioxidant activity (DPPH and ABTS^+^ scavenging rate) was well maintained. Overall, G5Fer achieved the best balance among acidity, bioactive compounds, and consumer preference. In conclusion, *gardenia* fruit represents a promising substrate for developing a functional, palatable kombucha-like beverage, aligning with the growing demand for value-added fermented products.

## 1. Introduction

*Gardenia jasminoides* Ellis is an evergreen shrub belonging to the Rubiaceae family that is native to Southern China. It was first introduced to Britain in the mid-18th century and has since become widely distributed across Asia and Europe with a long history of cultivation and application in China, Korea, and Japan [1]. The dried ripe fruits of *G. jasminoides* (Zhi-zi) possess both medicinal and edible properties and have been used in traditional Chinese medicine for over 2000 years [2]. According to the Shennong Bencao Jing (The Divine Farmer’s Materia Medica), *gardenia* fruits exert therapeutic effects such as reducing fire, relieving irritability, clearing heat, promoting diuresis, cooling blood, and removing toxins. When applied topically, they alleviate swelling and pain [3]. *Gardenia* fruits are characterized by high volatile oil content and abundant bioactive compounds such as iridoids (primarily geniposide), crocins, flavonoids, polyphenols, organic acids, and polysaccharides. These active substances synergistically contribute to strong hepatoprotective, choleretic, neuroprotective, anti-inflammatory, antioxidant, and metabolic-regulatory effects [4]. In the food industry, *gardenia* fruits are commonly processed into food coloring agents, including *gardenia* yellow and *gardenia* blue. Recently, *gardenia* oil was approved as a novel food ingredient by the National Health Commission of the People’s Republic of China. However, the current extraction technologies for its bioactive compounds suffer from low efficiency and high cost, and the natural bioactive components exhibit poor solubility and stability [2], which constrains their direct functional application and high-value-added development in the food industry. In addition, the *gardenia* fruit itself has an intensely bitter and astringent flavor along with a characteristic medicinal odor, which severely limits consumer acceptability and potential integration into food and nutraceutical products [5]. Microbial fermentation is considered an effective bioprocessing strategy to improve the nutritional and sensory properties of medicinal and edible homologous plant-based products, mainly through microbial metabolic processes [6,7].

Kombucha is an ancient fermented beverage produced by a symbiotic culture of acetic acid bacteria, yeast, and lactic acid bacteria within a cellulose membrane known as a SCOBY [8,9,10]. The traditional substrates for fermenting kombucha are primarily green and black tea infusions derived from *Camellia sinensis* leaves. Kombucha exhibits a complex chemical composition, including tea infusion-derived vitamins, minerals, amino acids, and polyphenols, as well as organic acids produced by microbial fermentation [11]. The chemical composition of kombucha is largely shaped by the raw substrates used, which has driven a growing interest in exploring alternative plant sources for its production. Among these, medicinal and edible homologous herbs have demonstrated particular promise, as their abundant bioactive compounds can not only impart unique chemical profiles and sensory attributes but also enhance the functional properties of the final beverage [8,12]. Recent studies have been carried out using other types of medicinal and edible homologous plants infusions as alternative substrates for making novel kombucha. For instance, kombucha fermentation of golden-flower tea and of honeysuckle-flower tea yielded high sensory scores. Particularly, fermentation with golden-flower tea residues markedly improved antioxidant activity (up to 2.83-fold) and total polyphenol content (3.48-fold) and increased several bioactive compounds, while *honeysuckle* tea residues had limited effects [13]. Another study found that supplementing green tea kombucha with 10–20% yam extract and fermenting for 5 days significantly changed its biochemical (lower Brix, higher organic acids and volatiles), microbial (*B. bruxellensis* dominant), and sensory (higher turbidity, fruity aroma, altered acidity) profiles [14]. Likewise, Wang et al. reported that kombucha fermentation of *Eucommia ulmoides* leaf resulted in slower sugar consumption and superior in vitro antioxidant activity, with increases in total polyphenols, geniposidic acid, pinoresinol diglucoside, and rutin but decreases in total flavonoids and chlorogenic acid [8].

Although extensive research has been conducted on kombucha fermentation using various alternative plant infusions, the use of *gardenia* fruit as a novel substrate remains unexplored in the scientific literature. Therefore, this study aimed to evaluate the feasibility of *gardenia* fruit infusion as an alternative medium for kombucha production. To this end, a comprehensive quality characterization of fermented *gardenia* fruit kombucha was conducted in terms of physicochemical parameters, non-targeted metabolites, antioxidant activities, and sensory acceptance. The findings will provide theoretical support and experimental data for the high-value utilization of *gardenia* fruit and the development of functional fermented beverages.

## 2. Materials and Methods

### 2.1. Reagents and Chemical Materials

Undecayed dried *gardenia* fruits (*Gardenia jasminoides* Ellis, Shan-Zhizi, average fruit diameter 1.5–2.0 cm, collected at the full-ripening stage with intense red-orange pigmentation) were obtained from Fujian Province, China. The kombucha starter culture was purchased from Jining Shanghao Jiapin Trading Co., Ltd. (Jining, Shandong, China). Sugar was obtained from a supermarket at Zhejiang A&F University (Hangzhou, Zhejiang, China). Folin–Ciocalteu’s phenol reagent, gallic acid, crocin I, citric acid, tartaric acid, succinic acid, L-malic acid, 2,2′-azino-bis (3-ethylbenzothiazoline-6-sulfonic acid) (ABTS^+^), and 1,1-diphenyl-2-picrylhydrazyl (DPPH) were purchased from Shanghai Yuanye Bio-Technology Co., Ltd. (Shanghai, China). Ethanol, concentrated sulfuric acid, acetic acid, sodium carbonate, and sodium acetate were purchased from Sinopharm Chemical Reagents Co., Ltd. (Shanghai, China). Acetonitrile was purchased from Shanghai Aladdin Biochemical Technology Co., Ltd. (Shanghai, China). The high-performance liquid chromatography (HPLC) analysis was performed on an Essentia LC-16 system (Shimadzu, Kyoto, Japan) equipped with an Aminex HPX-87H column (300 × 7.8 mm, 9 μm; Bio-Rad Laboratories, Hercules, CA, USA).

### 2.2. Activation of Kombucha Starter Culture

Kombucha starter culture was prepared according to a previous method [15] with some modifications. Briefly, 10 g of black tea and 100 g of sugar were added to 1 L of water, and the mixture was boiled at 100 °C for 15 min. After cooling, the tea infusion was filtered through a sterile sieve to remove tea leaves. The resulting tea–sugar solution was then inoculated with 2% (*v*/*v*) kombucha liquid and 30 g/L SCOBY culture. The mixture was covered with a clean cloth and allowed to ferment at 27 °C ± 2 °C for 8 days.

### 2.3. Kombucha Preparation Based on Gardenia Fruit Powder

Fresh *gardenia* fruits were initially oven-dried at 60 °C for 30 min to 1 h, after which the temperature was increased to 90 °C and maintained for approximately 36 h until the moisture content reached 8%. Subsequently, undecayed dried *gardenia* fruits were shelled and pulverized in a multi-functional grinder. The resulting powder was passed through an 80-mesh sieve to obtain *gardenia* fruit powder. Kombucha beverages were then prepared by adding the *gardenia* fruit powder at concentrations of 2.5%, 5.0%, 7.5%, 10.0%, and 12.5% (*w*/*v*) (corresponding to groups designated as G2.5Fer, G5Fer, G7.5Fer, G10Fer, and G12Fer, respectively). To guarantee the exact uniformity and reproducibility of the micro-ecology, each distinct batch was strictly inoculated with 2% (*v*/*v*) mature kombucha liquid and a precisely portioned 30 g/L intact SCOBY culture. These *gardenia* fruit kombucha samples were fermented simultaneously inside a digital incubator at 27 °C ± 2 °C for 8 days to maintain environmental consistency. During the fermentation process, samples were collected on days 0, 2, 4, 6, and 8 for subsequent analyses. All treatments and subsequent sample preparations were performed in strict biological triplicates (*n* = 3) to ensure statistical accuracy.

### 2.4. Total Titratable Acidity Analysis of Gardenia Fruit Kombucha

Total titratable acidity (expressed as acetic acid equivalents) was determined via acid-base titration with 0.1 mol/L NaOH solution [8]. Briefly, the kombucha fermentation broth was diluted 10-fold with purified water provided to analysis. The total acidity was then calculated using the following formula:

Total acid (g/L) = [c × (*V*_1_ − *V*_2_) × k × F]/m × 1000 (1)

where c is the concentration of the NaOH titrant; *V*_1_ and *V*_2_ are the titrant volumes of the sample and blank, respectively; k is the acid-specific molar mass conversion factor, F is the dilution factor; m is the sample mass; and 1000 is the conversion factor.

### 2.5. The Organic Acid Content Analysis of Gardenia Fruit Kombucha

The organic acid content was quantified using HPLC on a Essentia LC-16 system (Shimadzu, Kyoto, Japan) as previous method [14]. Filtered samples (0.22 µm) were injected (20 µL) into an Aminex HPX-87H column (300 × 7.8 mm, 9 µm) at 45 °C, with a mobile phase of 0.006 mol/L H_2_SO_4_ at 0.2 mL/min and detection at 205 nm. Standard curves were established using standard solutions of L-malic acid, citric, tartaric, and succinic acids. Linear regression was performed between the peak areas (y) of each organic acid and its corresponding concentrations (x, g/L) to establish a standard curve equation.

### 2.6. Total Polyphenol and Total Flavonoid Contents Analysis of Gardenia Fruit Kombucha

Total polyphenol content in *gardenia* fruit kombucha samples was analyzed using the modified Folin–Ciocalteu method, as described previously [16]. A standard curve (y = 0.1863x − 0.0013, R^2^ = 0.9981) was established using gallic acid (0.1 mg/mL). For the determination, 6 mL of diluted sample were first mixed with 1 mL of Folin–Ciocalteu reagent and allowed to stand in the dark for 4 min. Following the addition of 3 mL of sodium carbonate (75 g/L), the mixture was heated in a water bath at 40 °C for 2 h in dark conditions. The absorbance was measured at 765 nm, and the total polyphenol content of kombucha was quantified as milligrams of gallic acid equivalents per 100 mL of fermentation broth (mg GAE/100 mL).

Total flavonoid content was determined using a modified nitrite colorimetric method described previously [10]. Briefly, 1 mL of diluted *gardenia* fruit kombucha sample was mixed with 0.5 mL of 5.0% (*w*/*v*) sodium nitrite (NaNO_2_) solution and allowed to react for 6 min. Subsequently, 0.5 mL of 5.0% (*w*/*v*) aluminum nitrate (Al (NO_3_)_3_) solution were added, and the reaction continued for another 6 min. Then, 4 mL of 4% (*w*/*v*) sodium hydroxide (NaOH) solution were added, followed by dilution with 30% (*v*/*v*) ethanol to a final volume of 6 mL. After thorough mixing and a 15 min reaction period, the absorbance was measured at 510 nm. The total flavonoid content was quantified as rutin equivalents. It is worth noting that the Folin-Ciocalteu reagent exhibits cross-reactivity with certain reducing flavonoids and non-phenolic reducing substances (e.g., ascorbic acid) [17]. Therefore, the total polyphenol and total flavonoid contents in this study represent relative equivalent values based on their respective standard curves (gallic acid and rutin) rather than absolute gravimetric concentrations, which explains the apparent numerical variance between the two assays.

### 2.7. Crocin-I Content Analysis of Gardenia Fruit Kombucha

Crocin-I content was measured by HPLC using a Welth ODS-3 column (4.6 mm × 250 mm, 5 μm) at 440 nm, with an injection volume of 10 μL, column temperature of 35 °C, mobile phase A of acetic acid–ammonium acetate buffer (pH 4), and mobile phase B of acetonitrile. The gradient elution program was: 0–1 min (20% B), 1–8 min (20–60% B), 8–8.1 min (60–70% B), 8.1–13 min (70–98% B), 13–15 min (98–20% B), and 15–20 min (20% B). A crocin I standard curve was established as y = 55,690x – 44,464 (R^2^ = 0.9992), exhibiting good linearity over the range of 1–40 μg·mL^−1^. Sample results were expressed as crocin-I content (mg/g).

### 2.8. Antioxidant Activities Analysis of Gardenia Fruit Kombucha

DPPH radical scavenging activity was determined by the colorimetric method with some minor modifications [8]. The *gardenia* fruit kombucha sample (0.2 mL) or anhydrous ethanol (0.2 mL, blank) was mixed with 3.8 mL of a 0.2 mmol/L ethanol–DPPH solution. The mixture was then diluted with distilled water to a final volume of 5 mL and incubated in the dark for 30 min. After incubation, the absorbance was measured at 517 nm, and the DPPH radical scavenging activity was calculated using the following equation.

DPPH radical scavenging rate (%) = [(A_0_ − A_1_)/A_0_] × 100%

where A_0_ is the absorbance measured with anhydrous ethanol (blank), and A_1_ is the absorbance measured with the *gardenia* fruit kombucha sample.

ABTS^+^ radical scavenging activity was determined by the colorimetric method proposed by [10,16]. Briefly, equal volumes of 7 mmol/L ABTS solution and 2.45 mmol/L potassium persulfate (K_2_S_2_O_8_) solution were mixed and allowed to react in the dark at room temperature for 12–16 h to generate the ABTS^+^ radical stock solution. Before use, the stock solution was diluted with anhydrous ethanol to obtain an absorbance of 0.70 ± 0.02 at 734 nm, yielding the ABTS^+^ working solution. Subsequently, 0.1 mL of the *gardenia* fruit kombucha sample (or anhydrous ethanol as the blank) was thoroughly mixed with 3.9 mL of the ABTS^+^ working solution. After reacting in the dark for 10 min, the absorbance was measured at 734 nm. The ABTS radical scavenging rate (%) was calculated using the following formula:

ABTS^+^ radical scavenging rate (%) = [(A_0_ − A_1_)/A_0_] × 100%

where A_0_ is the absorbance measured with anhydrous ethanol (blank), and A_1_ is the absorbance measured with the *gardenia* fruit kombucha sample.

### 2.9. Non-Targeted Metabolomic Analysis of Gardenia Fruit Kombucha

Non-targeted metabolomic analysis of *gardenia* fruit kombucha samples was performed on an ultra-high-performance liquid chromatography system equipped with a Fourier transform mass spectrometer (UHPLC-Exploris 240, Thermo Fisher Scientific, Waltham, MA, USA). Briefly, 200 μL of the kombucha sample were accurately transferred into a 1.5 mL centrifuge tube, and 800 μL of extraction solution (methanol: acetonitrile = 1:1, *v*/*v*) containing an internal standard (0.02 mg/mL L-2-chlorophenylalanine) were added. The mixture was vortexed for 30 s, followed by low-temperature ultrasonic extraction at 5 °C and 40 kHz for 30 min. The samples were allowed to stand at −20 °C for 30 min and then centrifuged at 13,000 *g* and 4 °C for 15 min. The supernatant was collected and evaporated to dryness under a nitrogen stream. The residue was reconstituted in 120 μL of acetonitrile/water (1:1, *v*/*v*), vortexed for 30 s, and subjected to low-temperature ultrasonic extraction (5 °C, 40 kHz, 5 min) and a final centrifugation (13,000 *g*, 4 °C, 10 min). The supernatant was then transferred into autosampler vials with inserts for instrumental analysis. Meanwhile, 20 μL of supernatant from each sample were pooled to prepare quality-control (QC) samples, which were processed using the same procedure as the analytical samples. During the instrument run, one QC sample was injected after every 5–15 analytical runs to monitor the stability of the analytical process.

Chromatographic separation was performed on an ACQUITY UPLC HSS T3 column (100 mm × 2.1 mm i.d., 1.8 µm, Waters, Milford, MA, USA) with the column temperature set at 40 °C and an injection volume of 3 µL. Mobile phase A consisted of 95% water and 5% acetonitrile (containing 0.1% formic acid), and mobile phase B comprised 47.5% acetonitrile, 47.5% isopropanol, and 5% water (also containing 0.1% formic acid). The eluate from the column was detected via mass spectrometry using an electrospray ionization source, with signals acquired in both positive and negative ion-scanning modes. The mass scan range was set from 70 to 1050 m/z. The sheath gas flow rate was 60 arb, the auxiliary gas flow rate was 20 arb, the auxiliary gas heating temperature was 350 °C, and the capillary temperature was 320 °C. The spray voltages in positive and negative ion modes were set at +3400 V and −3000 V, respectively. The S-Lens voltage was 70. The data-dependent acquisition (DDA) mode with normalized collision energies (20, 40, 60 eV) was used to obtain tandem mass spectrometry information. The resolutions for MS1 and MS2 were set to 60,000 and 15,000, respectively.

The raw mass spectrometry data were imported into Progenesis QI v3.0 software for preprocessing to generate a data matrix containing retention time, m/z, and peak intensity. Metabolites were identified by matching against the HMDB, METLIN, and an in-house database (MS mass error < 10 ppm, combined with MS/MS matching scores). The identified raw data matrix was then preprocessed by removing missing values based on the 80% rule, filling missing values with the minimum detected value, performing total sum normalization, and removing variables with a relative standard deviation (RSD) > 30% in QC samples. Following log10 transformation, principal component analysis (PCA) and partial least-squares discriminant analysis (PLS-DA) were conducted. Differential metabolites were screened based on variable importance in projection (VIP > 1) from the OPLS-DA model combined with Student’s *t*-test (*p* < 0.05), followed by pathway annotation via the KEGG database and enrichment analysis using Fisher’s exact test. The complete dataset and curation metrics are now provided in the revised Supplementary Table S1.

### 2.10. Sensory Analysis of Gardenia Fruit Kombucha

The sensory evaluation was performed following a previously described method with minor modifications [16]. A sensory panel comprising 10 assessors (5 males and 5 females, aged 20–30 years) was recruited from Zhejiang A&F University. Panelists were selected according to ISO 8586 standards [18], based on their availability, lack of allergies to tea or *gardenia* fruit, and basic gustatory and olfactory sensitivity.

*Gardenia* fruit kombucha samples (20 mL each) were served in transparent cups randomly coded with three-digit numbers at room temperature. The cups were placed in sealed containers and assessed within 30 min by the trained panel. Each evaluation session took place in individual sensory booths under white light. A 1 min rest interval was provided between samples, during which panelists rinsed with purified water to eliminate carry-over effects. Each sample was evaluated in duplicate by each panelist.

Five attributes were evaluated (color, aroma, flavor, transparency, and overall acceptance) using a nine-point scale (1 = extremely unfavorable, 9 = extremely favorable). The detailed scoring criteria are provided in Supplementary Table S2. Chinese national regulations do not require ethical approval for sensory studies because: (a) consumer participation was purely consultative; (b) the results of the participation were directly applicable only to the specific study in question; and (c) the research involved drawing on consumers’ experiential knowledge for insights. Nevertheless, all participants approved and signed the informed consent to protect their rights and privacy, ensure voluntary participation, and allow withdrawal at any time.

### 2.11. Statistical Analysis

Data are expressed as the mean ± standard deviation (SD) and were plotted using Graphpad Prism 10.3.1 (GraphPad Software Inc., San Diego, CA, USA). Statistical significance was assessed using GraphPad Prism 10.3.1 or SPSS 27.0 (IBM SPSS, Armonk, NY, USA). Comparisons between two groups were performed using Student’s t-test, and multiple group comparisons were conducted using Tukey’s post-hoc test following two-way ANOVA. A *p*-value < 0.05 was considered statistically significant. The processing and analysis of non-targeted metabolomics data were conducted utilizing the proprietary cloud platform (https://www.majorbio.com/tools, accessed on 20 May 2025) of Shanghai Majorbio Bio-Pharm Technology Co., Ltd. (Shanghai, China).

## 3. Results and Discussion

### 3.1. Appearance of Gardenia Fruit Kombucha

**Figure 1:**
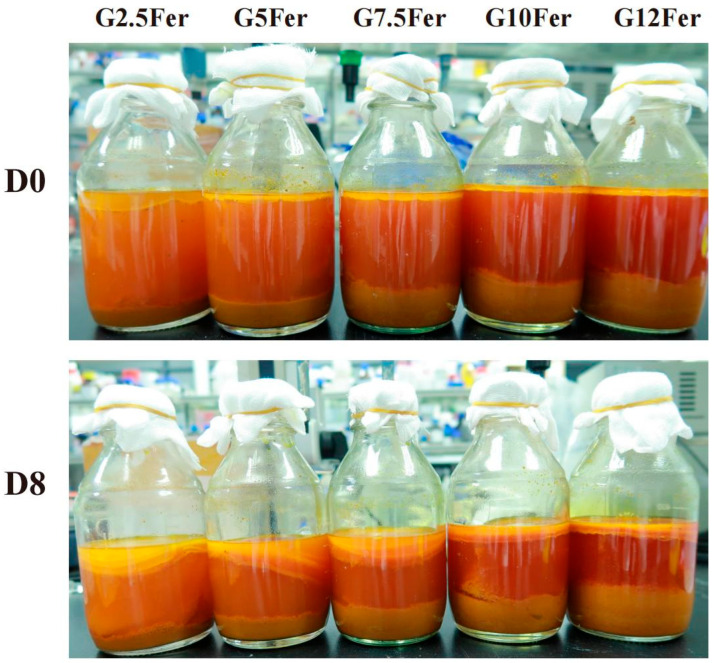
Visual morphology of bacterial cellulose membranes and the appearance of fermented beverages after 8 days of kombucha fermentation with varying concentrations of *gardenia* fruit powder. PreFer represents the unfermented baseline control. G2.5Fer, G5Fer, G7.5Fer, G10Fer, and G12Fer denote the kombucha formulations supplemented with 2.5%, 5.0%, 7.5%, 10.0%, and 12.5% (*w*/*v*) of *gardenia* fruit powder, respectively. D0: Initial fermentation stage at day 0; D8: Final fermentation stage at day 8.

In this study, *gardenia* fruit was fermented to develop a novel alternative kombucha-like beverage. Due to the high content of *gardenia* yellow pigment in *gardenia* fruit, the color intensity of the *gardenia* fruit kombucha samples increased with the amount of *gardenia* fruit added, shifting from bright golden yellow to dark brown, and even to reddish-brown both at the initial stage (day 0) and after 8 days of fermentation (day 8) (Figure 1). On day 8, a thick SCOBY–cellulose layer formed on the surface. Among the groups, G5Fer developed thicker pellicles compared to G2.5Fer, G7.5Fer, G10Fer, and G12Fer. This SCOBY–cellulose is produced by acetic acid bacteria, while ethanol generated by yeast promotes bacterial growth, thereby stimulating further cellulose and acetic acid synthesis [16]. These macroscopic pellicle developments provide direct visual insight into the underlying compositional remodeling during fermentation [19]. Notably, the phenotypic differences in pellicle formation exhibited a clear dose-dependent pattern. An optimal initial substrate concentration (5.0% *w*/*v*) promoted the most vigorous pellicle growth, which expanded and partially submerged into the liquid phase by Day 8. In contrast, higher substrate dosages (7.5–12.5% *w*/*v*) exerted a pronounced inhibitory effect on pellicle development. For these high-dose groups, the surface pellicles remained visibly thinner and structurally constrained at Day 8, while substantial undissolved substrate residues persistently accumulated at the vessel bottoms, closely resembling their initial Day 0 physical state.

### 3.2. Variations in Total Titratable Acidity of Gardenia Fruit Kombucha

**Figure 2:**
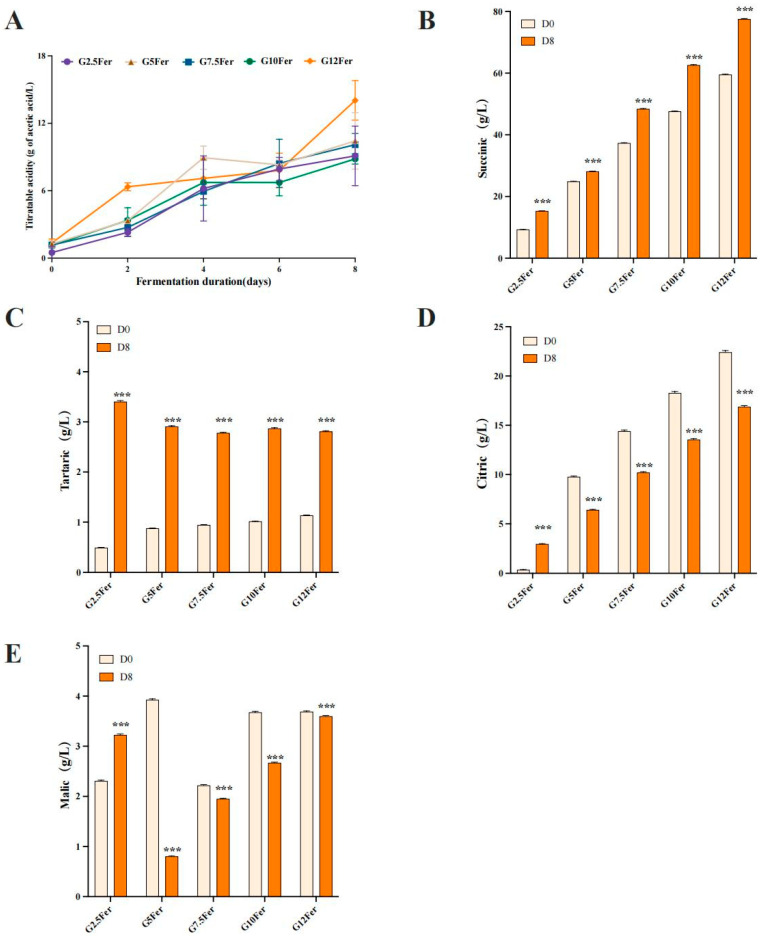
Variations in total titratable acidity (TTA) and high-performance liquid chromatography (HPLC) quantitative profiles of organic acids in *gardenia* fruit kombucha beverages between the initial (Day 0) and final (Day 8) fermentation stages. (**A**) Evolution of total titratable acidity (expressed as g/L); (**B**) succinic acid content; (**C**) tartaric acid content; (**D**) citric acid content; and (**E**) malic acid content (all organic acids in panels B–E are expressed as mg/100 mL). D0: Initial fermentation stage at day 0; D8: Final fermentation stage at day 8. G2.5Fer, G5Fer, G7.5Fer, G10Fer, and G12Fer represent the fermented kombucha formulations fortified with 2.5%, 5.0%, 7.5%, 10.0%, and 12.5% (*w*/*v*) of *gardenia* fruit powder, respectively. All bars represent the mean ± SD deviations (*n* = 3). *** means *p* < 0.001 comparation between D8 and D0 within the same *gardenia* fruit concentration.

Acidity is one of the distinctive flavor characteristics and essential quality attributes of kombucha [11,16]. A continuous rise in total titratable acidity (expressed as acetic acid equivalents, Figure 2A) was observed in all fermentation samples, reflecting the vigorous microbial acid-producing metabolic activities in the fermentation system [8], with day 4 being the active period for acid production. Specifically, the average titratable acidity in the G2.5Fer group increased from 0.50 g/L (day 0) to 6.20 g/L (day 4, *p* < 0.05), the G5Fer group from 1.27 g/L (day 0) to 8.93 g/L (day 4, *p* < 0.05), the G7.5Fer group from 1.17 g/L (day 0) to 5.93 g/L (day 4, *p* < 0.05), the G10Fer group from 1.13 g/L (day 0) to 6.73 g/L (day 4, *p* < 0.05), and the G12Fer group from 1.35 g/L (day 0) to 7.10 g/L (day 4, *p* < 0.05). At the end of fermentation (day 8), the total titratable acidity in all fermentation samples reached its peak, with values of 9.10 g/L (G2.5Fer), 10.43 g/L (G5Fer), 10.10 g/L (G7.5Fer), 8.83 g/L (G10Fer), and 14.03 g/L (G12Fer). Among them, the G12Fer group exhibited the highest final acidity and the greatest increase, followed by the G5Fer group, with no significant difference observed between these two groups.

This progressive accumulation of organic acids in the *gardenia* fruit kombucha was primarily driven by the cooperative metabolic activities of yeast and acetic acid bacteria. This metabolic efficiency and the resulting high acidity levels (ranging from 8.83 to 14.03 g/L) represent a substantial enhancement compared to alternative plant-based kombucha analogs reported in recent literature. For instance, in passion fruit and apple kombucha-like beverages, the maximum titratable acidity values only reached 1.05% and 0.65% (approximately 10.50 g/L and 6.50 g/L) by the end of fermentation, respectively, whereas traditional tea kombucha exhibited an even lower organic acid conversion [20].

In comparison with traditional and commercial kombucha products, which typically exhibit a titratable acidity threshold of 4.0–9.0 g/L for optimal palatability [21,22], the G12Fer group showed excessive acid accumulation, reaching 14.03 g/L. Although this high-dose fortification maximizes the extraction of specific functional bioactives, the resultant pungent sourness severely compromises the balanced sensory profile essential for commercial beverage development. This empirical limitation clearly establishes the G5Fer formulation as the optimal candidate for balancing industrial scalability, sensory acceptability, and biofunctional efficacy.

Such pronounced variations across different botanical substrates reinforce the principle that the dynamic biosynthesis of organic acids during kombucha fermentation is strictly governed by the specific carbon and nitrogen profiles liberated from the raw matrix, which directly modulate the synergistic metabolic flux within the yeast and bacterial communities [23].

Mechanistically, the pronounced increase in total titratable acidity observed in the G5Fer group aligns with its thicker bacterial cellulose pellicle (Figure 1), suggesting that an accelerated symbiotic proliferation of the starter culture effectively promotes organic acid biosynthesis, thereby establishing a hostile environment against potential pathogenic contaminants and ensuring microbiological stability [16].

### 3.3. Variations in Organic Acids of Gardenia Fruit Kombucha

The observed increase in total titratable acidity can be attributed to the organic acids generated during microbial metabolism [24]. During kombucha fermentation, yeast converts sugars into ethanol and CO_2_ via the glycolysis pathway [25], while acetic acid bacteria further transform ethanol into acetic and tartaric acids. Over time, additional organic acids such as malic and succinic acids also accumulate [16].

#### 3.3.1. Inherent Metabolic Dynamics of Organic Acids During Fermentation

To evaluate the inherent fermentation characteristics driven by the symbiotic culture of bacteria and yeast (SCOBY), the dynamic variations of individual organic acids from day 0 to day 8 were monitored via HPLC analysis (Figure 2B–E). Compared to the unfermented samples (day 0), the contents of succinic and tartaric acids in all *gardenia* fruit kombucha samples dramatically increased at the end of fermentation (day 8). Specifically, after 8 d of fermentation, the succinic acid content in the respective fermentation broths reached 15.36 g/L, 28.23 g/L, 48.50 g/L, 62.73 g/L, and 77.67 g/L, representing elevations of 64.82%, 13.02%, 29.63%, 31.56%, and 30.23% compared to their initial stages (Figure 2B). Regarding tartaric acid accumulation, the contents on day 8 were 3.41 g/L (G2.5Fer), 2.92 g/L (G5Fer), 2.79 g/L (G7.5Fer), 2.88 g/L (G10Fer), and 2.82 g/L (G12Fer), which increased significantly by 584.03%, 230.35%, 193.16%, 182.51%, and 147.54% compared to the initial values, respectively (Figure 2C).

Conversely, the contents of citric and malic acids (except for the G2.5Fer group) exhibited a significant decrease by the end of fermentation. As shown in Figure 2D, the citric acid values changed from initial levels to 0.30 g/L, 0.65 g/L, 1.03 g/L, 1.36 g/L, and 1.70 g/L on day 8. Meanwhile, the malic acid values shifted from their day 0 levels to 3.22 g/L, 0.81 g/L, 1.95 g/L, 2.67 g/L, and 3.60 g/L, respectively, on day 8 (Figure 2E).

Biochemically, citric acid and malic acid serve as key intermediates of the tricarboxylic acid cycle (TCA), and they are preferentially utilized during the proliferation and metabolism of the kombucha microbial consortium. For example, *Acetobacter* and *Komagataeibacter*, which possess a complete TCA cycle, can directly oxidize these two organic acids, whereas *Gluconobacter* lacks the key enzymes and cannot complete this process [26]. Meanwhile, lactic acid bacteria convert citric acid into acetic acid via citrate cleavage, and certain strains can also generate succinic acid through the malate–fumarate pathway [27]. In addition, malic acid may be fermented by lactic acid bacteria and converted into lactic acid, thereby subjecting yeast to lactic acid stress and further inhibiting the TCA cycle-driven synthesis of malic acid [28]. Therefore, the decrease in citric acid and malic acid during fermentation reflects their breakdown as readily available carbon sources by the SCOBY consortium and their directional conversion into functional organic acids such as acetic acid and succinic acid [29].

Correspondingly, decreases in citric and malic acids followed the same trend as the changes in total titratable acidity and succinic acid.

#### 3.3.2. Direct Chemical Impacts and Dosage Effects of Gardenia Fruit Powder Addition

Distinct from microbial metabolic fluctuations over time, the addition of *gardenia* fruit powder exerted a direct, dose-dependent impact on the baseline concentrations of specific organic acids. Notably, the contents of succinic acid and citric acid significantly increased with the increasing addition of *gardenia* fruit powder both at the initial unfermented stage (day 0) and at the end of fermentation (day 8). Initially (day 0), the succinic acid content scaled proportionally with the powder dosage, recorded at 9.32 g/L (G2.5Fer), 24.98 g/L (G5Fer), 37.42 g/L (G7.5Fer), 47.69 g/L (G10Fer), and 59.65 g/L (G12Fer), respectively (Figure 2B). A similar direct input effect was observed for citric acid on day 0, with initial values for the G2.5Fer, G5Fer, G7.5Fer, G10Fer, and G12Fer groups measured at 0.04 g/L, 0.98 g/L, 1.45 g/L, 1.84 g/L, and 2.25 g/L, respectively (Figure 2D). Furthermore, the initial malic acid values also reflected this exogenous contribution, starting at 2.31 g/L, 3.92 g/L, 2.22 g/L, 3.68 g/L, and 3.69 g/L, respectively (Figure 2E).

This phenomenon is closely associated with the chemical matrix of the raw material. Previous studies have confirmed that succinic acid accumulates progressively during the development and maturation of *gardenia* fruit, representing one of its inherent organic acids [30]. Furthermore, Xiao et al. reported that organic acids constitute a major class of active compounds in *gardenia* fruit [31]. Therefore, the introduction of *gardenia* fruit powder directly enriches the initial beverage matrix with these plant-derived acids. Concurrently, it provides abundant metabolic substrates for the microorganisms, thereby continuously promoting and sustaining higher absolute accumulations of succinic acid and other organic acids throughout the kombucha fermentation system.

Ultimately, the dynamic equilibrium among different organic acids, characterized by reciprocal variations in their concentrations, prevents an excessive elevation of acidity that would otherwise exacerbate sourness and consequently compromise the sensory score of the sample [32].

### 3.4. Variations in Total Polyphenol of Gardenia Fruit Kombucha

**Figure 3:**
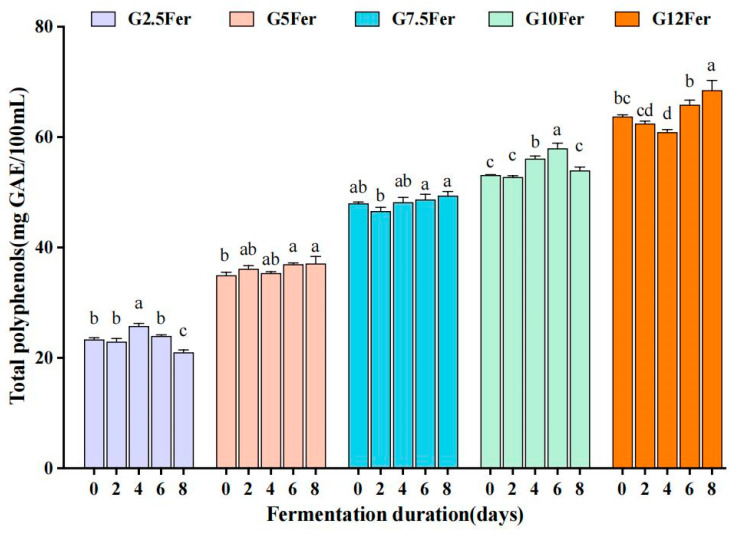
Dynamic evolution of total phenolic content (TPC) in *gardenia* fruit kombucha beverages prepared with varying substrate mass fractions over an 8-day fermentation period. TPC values are quantified and expressed as milligrams of gallic acid equivalents per 100 mL (mg GAE/100 mL) of the beverage. G2.5Fer, G5Fer, G7.5Fer, G10Fer, and G12Fer represent the fermented cohorts fortified with 2.5%, 5.0%, 7.5%, 10.0%, and 12.5% (*w*/*v*) of *gardenia* fruit powder, respectively. All data points are visualized as bars representing the means ± SD (*n* = 3). Different lowercase letters (a–d) indexed above the bars within the same *gardenia* fruit powder concentration indicate statistically significant differences across different fermentation days (*p* < 0.05) based on Tukey’s post-hoc test.

To evaluate the functional properties of the fermented *gardenia* fruit kombucha, the dynamic changes in total polyphenols, total flavonoids, and Crocin I were quantified. As shown in Figure 3, the total polyphenol content in *gardenia* fruit kombucha of G2.5Fer and G10Fer groups exhibited an initial increase, followed by a decline during fermentation. The total polyphenol content in the G2.5Fer group significantly increased from 23.34 mg GAE/100 mL to 25.78 mg GAE/100 mL (*p* < 0.05, day 4 vs. day 0), then continuously decreased to 21.00 mg GAE/100 mL on day 8 (*p* < 0.05, vs. day 0), representing a reduction of 10.0% compared to the initial value (day 0). In the G10Fer group, the initial total polyphenol content was 53.14 mg GAE/100 mL. On days 4 and 6 of fermentation, it significantly increased to 56.13 mg GAE/100 mL (*p* < 0.05) and 57.97 mg GAE/100 mL (*p* < 0.05), respectively, and then decreased to 53.99 mg GAE/100 mL on day 8, approaching the initial level. This initial increase may be attributed to the generation of aglycone flavonoids, a class of plant polyphenols widely acknowledged for their comparatively high chemical activity [33]. The subsequent decline, however, might result from the release of polyphenol oxidase and other oxidases during the later stages of fermentation, similar to what is observed in kombucha based on *Moringa oleifera* [7], whereas the total polyphenol content in the G7.5Fer group showed no significant difference from the initial content at any fermentation time point.

Notably, the G5Fer group showed a general increasing trend with fermentation time. The initial value was 34.98 mg GAE/100 mL, which increased to 36.14 mg GAE/100 mL, 35.38 mg GAE/100 mL, 36.98 mg GAE/100 mL, and 37.12 mg GAE/100 mL on fermentation days 2, 4, 6, and 8, respectively, with the increases on day 6 (*p* < 0.05) and day 8 (*p* < 0.05) being statistically significant. This observation is consistent with previous findings that kombucha fermentation based on *African mustard* leaves [34] and *Eucommia ulmoides* leaf [8] led to a sustained increase in total polyphenols content relative to unfermented leaves beverage. The increase in total polyphenol content during kombucha fermentation is attributed to the production of various enzymes (e.g., cellulase, glucanase, xylanase, pectinase, and glucosidase). These enzymes break down large molecules into smaller polyphenol monomers [35] and degrade plant cell walls, releasing insoluble bound phenols as soluble phenols [36].

In contrast, the total polyphenol content in the G12Fer group exhibited an initial decrease followed by an increase during fermentation. It significantly decreased from 63.74 mg GAE/100 mL to 60.91 mg GAE/100 mL (a reduction of 4.44%, *p* < 0.05, day 4 vs. day 0), and then gradually and significantly increased, reaching a peak value of 68.52 mg GAE/100 mL on day 8 (*p* < 0.05, vs. day 0). This pattern of first decreasing and then increasing is analogous to that observed for a novel kombucha-fermented *Sargassum fusiforme* beverage [37] and is attributed to the early-stage microbial utilization and enzymatic degradation of free phenolics, causing a temporary decrease, followed by the late-stage hydrolysis of bound phenolics (e.g., chlorogenic acid glycosides, catechin esters) into free phenolics via secreted glycosidases and cellulases, leading to a subsequent increase [38].

This enrichment strategy alters the initial chemical profile relative to traditional substrates. Standard black tea kombucha fermented under typical conditions yields a benchmark TPC of approximately 469.04 mg GAE/L, equivalent to 46.90 mg GAE/100 mL [37]. In this study, the unfermented baseline values of the *gardenia*-infused broths ranged from 23.34 to 63.74 mg GAE/100 mL. This indicates that adding *gardenia* fruit powder at appropriate concentrations can effectively elevate the initial phenolic content above that of conventional black tea kombucha. Furthermore, while black tea kombucha remains a rich source of antioxidants, its baseline profile generally presents a relatively stable or marginally fluctuating pattern, influenced by the specific SCOBY composition. Optimized incorporation of *gardenia* fruit offers a reliable method to substantially enhance total polyphenol concentrations, thereby maximizing the nutritional value and health-promoting efficacy of the final functional beverage.

Meanwhile, as indicated by the above results, the total polyphenol content in kombucha samples increased in a dose-dependent manner with the increasing amount of *gardenia* fruit powder added, demonstrating that *gardenia* fruit is the main source of phenolic compounds in the fermentation broth [39]. The ´previous study has revealed that *gardenia* fruit is rich in various bioactive components, including iridoid glycosides (e.g., geniposide), flavonoids (e.g., rutin, quercetin), and phenolic acids (e.g., chlorogenic acid, dicaffeoylquinic acid) [4]. Therefore, utilizing an appropriate dose of *gardenia* fruit for kombucha preparation could increase total polyphenols levels, thereby enhancing the nutritional value and potential health benefits of the beverage.

### 3.5. Variations in Total Flavonoid of Gardenia Fruit Kombucha

**Figure 4:**
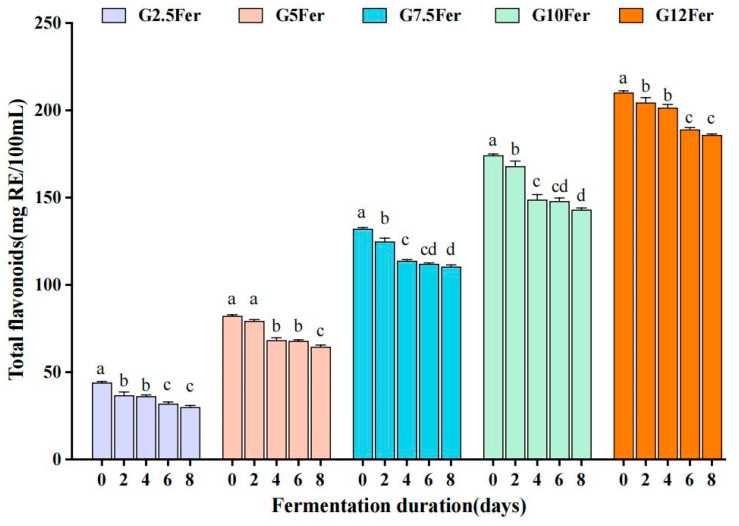
Dynamic variations in total flavonoid content (TFC) of *gardenia* fruit kombucha beverages prepared with varying substrate mass fractions over an 8-day fermentation period. TFC values are quantified spectrophotometrically and expressed as milligrams of rutin equivalents per 100 mL (mg RE/100 mL) of the beverage. G2.5Fer, G5Fer, G7.5Fer, G10Fer, and G12Fer represent the fermented cohorts fortified with 2.5%, 5.0%, 7.5%, 10.0%, and 12.5% (*w*/*v*) of *gardenia* fruit powder, respectively. All data points are visualized as bars representing the means ± SD deviations (*n* = 3). Different lowercase letters (a–d) indexed above the bars within the same *gardenia* fruit powder concentration indicate statistically significant differences across different fermentation days (*p* < 0.05) based on Tukey’s post-hoc test.

Throughout the fermentation period, the total flavonoid concentrations in all *gardenia* fruit kombucha samples showed a continuous declination compared to the initial stage (day 0). Initially, the total flavonoid content in *gardenia* fruit kombucha samples were 44.16 mg RE/100 mL (G2.5Fer), 82.31 mg RE/100 mL (G5Fer), 132.27 mg RE/100 mL (G7.5Fer), 174.37 mg RE/100 mL (G10Fer), and 210.24 mg RE/100 mL (G12Fer), respectively. After 8 d of fermentation, the total flavonoid content in the respective fermentation broths was 30.07 mg RE/100 mL, 64.59 mg RE/100 mL, 110.58 mg RE/100 mL, 143.16 mg RE/100 mL, and 185.94 mg RE/100 mL, representing the overall reduction of 31.86%, 21.53%, 16.40%, 17.89%, and 11.56% compared to the initial stage (Figure 4).

Consistent with the total polyphenol content, the total flavonoid content in kombucha samples exhibited a dose-dependent increase in response to *gardenia* fruit powder addition, revealing that *gardenia* fruit serves as the primary source of polyphenols/flavonoids in the fermentation broth [39]. Moreover, our findings are consistent with previous research. Liang et al. observed a 38.98% decrease in flavonol glycosides and a significant negative correlation between microbial abundance and kaempferol-3-glucoside content, demonstrating the flavonoid-metabolizing capacity of kombucha microbes [40]. Wei et al. further identified that enzymes such as NADPH dehydrogenase 3 may be involved in catechin degradation [41]. In the present study, microbes likely utilize *gardenia* fruit flavonoids as direct metabolic substrates, resulting in a continuous decline in their content during fermentation. Additionally, recent research has shown that the total flavonoid content in three types of kombucha (black tea kombucha, black tea-red yeast rice mixed kombucha, and red yeast rice kombucha) exhibited a declining trend as fermentation time extended [16]. This phenomenon could be attributed to that, in the kombucha fermentation system, yeasts and acetic acid bacteria secrete polyphenol oxidase, laccase, β-glucosidase, and other enzymes that preferentially convert high-concentration flavonoid compounds into small-molecule phenolic acids (e.g., gallic acid, protocatechuic acid) or directly utilize them as carbon sources, thereby leading to a continuous decrease in total flavonoid content [42]. Furthermore, the structural instability of flavonoids [8] and the cell walls or metabolites of mixed microorganisms may form complexes with flavonoids, thereby further reducing their detectable content [43].

### 3.6. Variations in Crocin-I of Gardenia Fruit Kombucha

**Figure 5:**
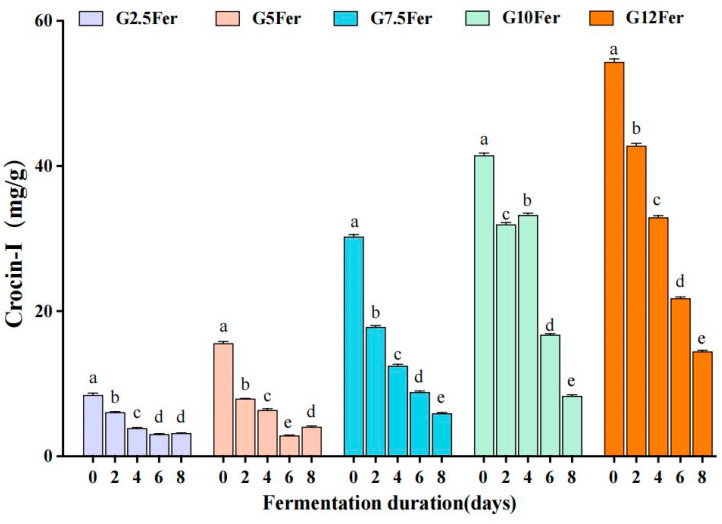
Dynamic evolution of crocin-I content in *Gardenia* fruit kombucha beverages prepared with varying substrate mass fractions over an 8-day fermentation period. Crocin-I content is quantified and expressed as milligrams per gram (mg/g) of the dry weight substrate. PreFer denotes the unfermented baseline control group (0 days); G2.5Fer, G5Fer, G7.5Fer, G10Fer, and G12Fer represent the fermented cohorts fortified with 2.5%, 5.0%, 7.5%, 10.0%, and 12.5% (*w*/*v*) of *gardenia* fruit powder, respectively. All data points are visualized as bars representing the means ± SD. Different lowercase letters (a–e) indexed above the bars within the same *gardenia* fruit powder concentration indicate statistically significant differences across different fermentation days (*p* < 0.05) based on Tukey’s post-hoc test.

The dynamic monitoring results of crocin-I content in the *gardenia* fruit kombucha samples are shown in Figure 5. At the initial unfermented stage (day 0), crocin-I, as one of the main active components of *gardenia* fruit [44], significantly increased with the amount of *gardenia* fruit added. The initial contents in each group were 8.47 mg/g (G2.5Fer), 15.58 mg/g (G5Fer), 30.28 mg/g (G7.5Fer), 41.49 mg/g (G10Fer), and 54.35 mg/g (G12Fer), respectively. As fermentation progressed, all groups exhibited a decreasing trend in crocin-I content, with overall reductions of 62.10% (G2.5Fer), 73.70% (G5Fer), 80.35% (G7.5Fer), 79.90% (G10Fer), and 73.40% (G12Fer), respectively, except for the G5Fer group. In the G5Fer group, the crocin-I content continuously decreased to a minimum of 2.90 mg/g during the first 6 days but then showed an upward trend on day 8, reaching a final content of 4.09 mg/g. The decrease in crocin-I content may be attributed to the efficient hydrolysis of the glycosidic bonds in the crocin molecule by β-glucosidase secreted by the kombucha microbiota, converting crocin-I into crocetin or smaller molecular metabolites, thereby leading to the reduction in its content [44,45].

### 3.7. Antioxidant Properties of Gardenia Fruit Kombucha

**Figure 6:**
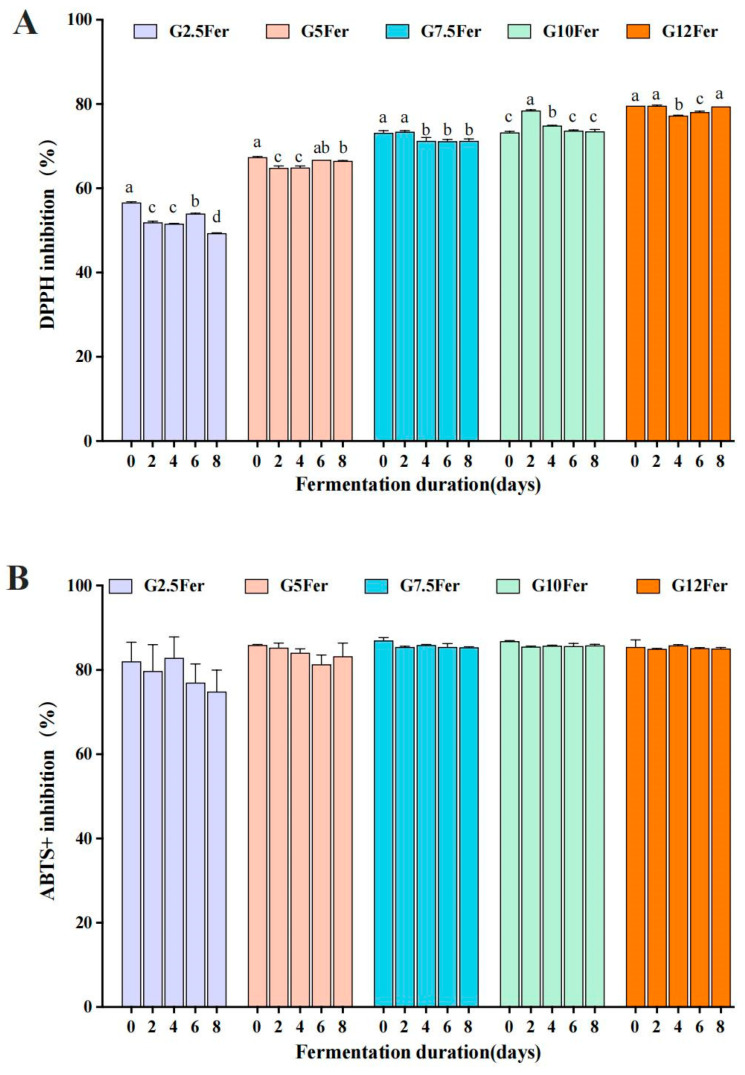
Evaluation of the in vitro antioxidant capacities of *Gardenia* fruit kombucha beverages prepared with varying substrate mass fractions over an 8-day fermentation period. (**A**) DPPH radical scavenging inhibition percentage; (**B**) ABST^+^ radical scavenging inhibition percentage (both indices are expressed as %). PreFer denotes the unfermented baseline control group (0 days); G2.5Fer, G5Fer, G7.5Fer, G10Fer, and G12Fer represent the fermented cohorts fortified with 2.5%, 5.0%, 7.5%, 10.0%, and 12.5% (*w*/*v*) of *gardenia* fruit powder, respectively. All data points are visualized as bars representing the means ± SD (*n* = 3). Different lowercase letters (a–d) indexed above the bars within the same *gardenia* fruit powder concentration indicate statistically significant differences across different fermentation days (*p* < 0.05) based on Tukey’s post-hoc test.

The abundant bioactive compounds (organic acids, polyphenols, and flavonoids) in fermentation broth are closely associated with its in vitro antioxidant activity, which serves as an important indicator for evaluating the functional activity of *gardenia* fruit kombucha [8,11]. The antioxidant activities were evaluated using DPPH and ABTS^+^ radical scavenging activity (Figure 6). The initial DPPH radical scavenging rates of the unfermented *gardenia* fruit kombucha were determined to be 56.66% (G2.5Fer), 67.42% (G5Fer), 73.19% (G7.5Fer), 73.27% (G10Fer), and 79.61% (G12Fer), respectively (Figure 6A).

Strikingly, compared with the baseline of a traditional black tea kombucha control reported by Sabatini et al., a standard unfortified black tea formulation yielded a baseline DPPH radical scavenging rate of approximately 20% [45]. Even the lowest dosage group (G2.5Fer) in the present study maintained a post-fermentation DPPH scavenging rate of 49.36%, which is more than double the capacity of unfortified tea reference. Meanwhile, the DPPH scavenging rates of the other groups (G5Fer, G7.5Fer, G10Fer, and G12Fer) remained relatively stable during fermentation, ranging between 65% and 80%, with no significant decrease compared to the unfermented samples. This sustained high-level DPPH scavenging activity (65–80%) clearly demonstrates that the exogenous *gardenia* components act as a powerful booster to the overall antioxidant profile, far transcending the natural constraints of basic tea polyphenols and confirming the unique value of this botanical fortification strategy. Overall, this suggests that kombucha fermentation does not compromise the antioxidant activity of *gardenia* fruit.

In the ABTS assay, the initial average scavenging rates of all the unfermented *gardenia* fruit kombucha samples exceeded 80%, namely: 82.08% (G2.5Fer), 85.94% (G5Fer), 87.04% (G7.5Fer), 86.84% (G10Fer), and 85.48% (G12Fer), respectively (Figure 6B). During the 8-day fermentation process, the scavenging rates of the G2.5Fer group decreased from 56.66% to 49.36% (DPPH) and from 82.08% to 74.91% (ABTS), respectively, whereas those of the other groups remained relatively stable. Specifically, during fermentation, the DPPH and ABTS scavenging rates of G5Fer, G7.5Fer, G10Fer, and G12Fer groups remained between 65–80% and 80–85%, respectively, with no significant decrease compared to the unfermented samples, suggesting that kombucha fermentation does not compromise the antioxidant activity of *gardenia* fruit.

Similarly, the DPPH and ABTS^+^ radical scavenging rates showed an increasing trend with the increase in *gardenia* fruit dosage (from 2.5% to 7.5%), a result consistent with the above observation that total polyphenols and flavonoid contents showed a dose-dependent increase in response to *gardenia* fruit powder addition (Figure 3 and Figure 4). The bioactive iridoid glycosides, flavonoid, and phenolic acids in *gardenia* fruit exert significant antioxidant activity by providing phenolic hydroxyl groups to combine with DPPH radicals, and their antioxidant activity is positively correlated with the dosage [46]. However, with a further increase in the amount of *gardenia* fruit powder added (≥7.5%), the ABTS^+^ scavenging rate did not increase significantly. The possible reason is that polyphenols and flavonoids are not the only factors responsible for the enhanced antioxidant capacity of the samples; the generation of organic acids, small-molecule active peptides, and other compounds during fermentation may also alter their hydroxyl radical scavenging ability [37,47]. In this study, the fact that changes in total polyphenols and flavonoid contents did not lead to a significant alteration in antioxidant activity may be attributable to the dynamic equilibrium among different organic acids.

### 3.8. Non-Targeted Metabolomics Analysis of Gardenia Fruit Kombucha

We further employed non-targeted metabolomics techniques to identify and quantify specific phenolic and flavonoid compounds, providing insights into their dynamic changes during fermentation and facilitating a better understanding of the metabolic pathways and biological functions of these compounds. Based on the physicochemical and antioxidant properties, *gardenia* fruit kombucha at the initial unfermented stage (designated as PreFer group; Day 0, prepared with 5.0% *gardenia* fruit powder addition prior to fermentation) and the G5Fer and G12Fer groups (collected at the end of fermentation on Day 8) were selected to determine. To comprehensively understand the metabolite composition and structural characteristics throughout the fermentation of *gardenia* fruit kombucha, raw data from the samples were preprocessed under both positive and negative ionization modes. Notably, 1518 compounds were putatively identified in positive ion mode and 1682 compounds in the negative ion mode. Of these, the number of metabolites annotated to the KEGG database was 465 for the positive ion group and 595 for the negative ion group.

**Figure 7:**
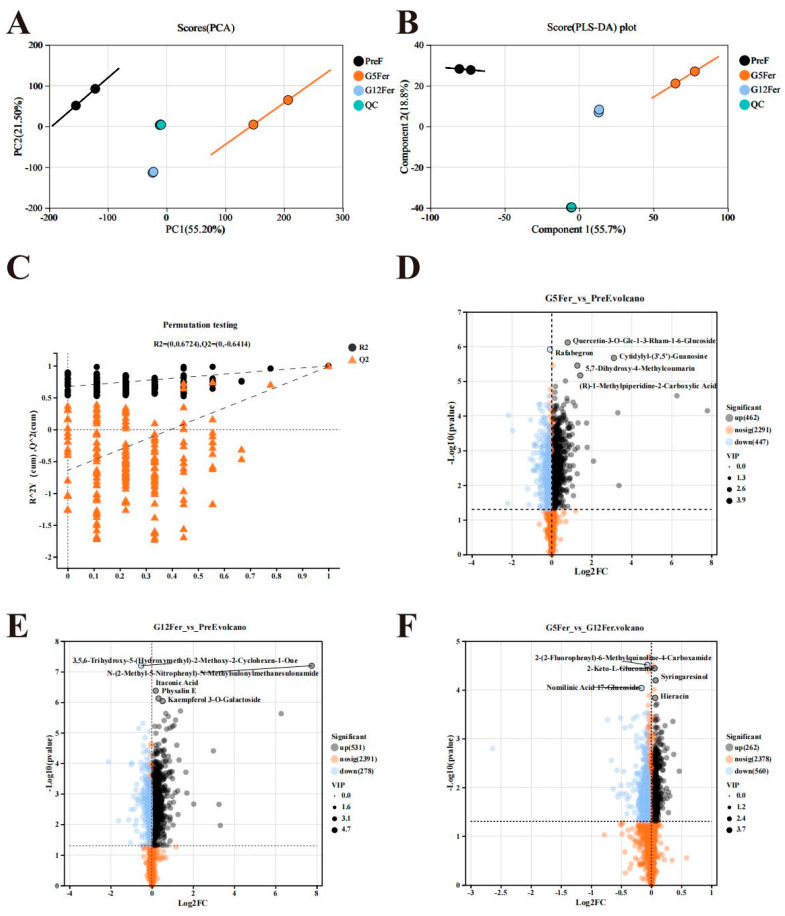
Non-targeted metabolomics analysis of differential metabolites between *gardenia* fruit kombucha at concentrations of 5.0% (G5Fer) and 12.5% (G12Fer) and initial unfermented kombucha at day 0 (Prefer). Results of (**A**) Principal component analysis (PCA) and (**B**) partial least-squares discriminant analysis (PLS-DA) of quality-control samples (QC) prefers G5Fer and G12Fer groups. (**C**) The permutation test charts for PLS-DA model. Volcano plots of differentially accumulated metabolites between fermentation groups: (**D**) G5Fer group vs. Prefer group; (**E**) G12Fer group vs. Prefer group; and (**F**) G5Fer group vs. G12Fer group.

As shown in the principal component analysis (PCA) score plot (Figure 7A), the replicate samples within each fermentation group and the quality-control (QC) samples clustered closely together, demonstrating good data reproducibility and reliability. In addition, the first two principal components (PC1 and PC2) explained 55.20% and 21.50% of the total variance, respectively. Samples from the PreFer, G5Fer, and G12Fer groups exhibited a clear separation trend along the PC1 axis. To further assess intergroup differences, partial least-squares discriminant analysis (PLS-DA) was performed. The PLS-DA score plot (Figure 7B) revealed a clear and complete separation of the PreFer, G5Fer, and G12Fer groups along the PC1 axis, indicating that kombucha fermentation profoundly reshaped the metabolic profile of *gardenia* fruit. The robustness of the PLS-DA model was validated through a permutation test (Figure 7C). The Q^2^ regression line intersected the Y-axis at a value corresponding to *p* < 0.05, with R^2^ and Q^2^ values of 0.999 and 0.987, respectively, confirming that the model was reliable and free from overfitting.

Based on these validations, differential metabolites (DEMs) between each comparation group were tentatively annotated using the criteria of variable importance in projection (VIP) > 1 and *p* < 0.05. In strict accordance with the Metabolomics Standards Initiative (MSI) reporting standards, all compounds discussed in this study are putatively annotated at MSI Level 2/3 based on the matching of accurate precursor masses (*m*/*z* values), isotopic distributions, and MS/MS fragmentation spectra against online spectral databases (Supplementary Table S3). Crucially, these featured compounds were purely assigned through spectral library matching and are thus tentatively annotated rather than unequivocally identified due to the lack of verification against authentic chemical reference standards.

The distribution patterns of these DEMs were visualized via volcano plots (Figure 7D–F). Between the G5Fer and PreFer groups, 909 differential metabolites were identified, comprising 462 upregulated and 447 downregulated compounds. Notably, the levels of quercetin-3-O-glucosyl-1-3-rhamnosyl-1-6-glucoside, refabegron, and cytidylyl-(3′,5′)-guanosine were significantly increased (Figure 7D). In the comparison between G12Fer and PreFer groups, 809 differential metabolites were detected, including 531 upregulated and 278 downregulated metabolites, with substantial increases in itaconic acid, physalin E, and kaempferol-3-O-glucoside/galactoside (Figure 7E). When comparing G5Fer with G12Fer, 822 differential metabolites were identified (262 upregulated and 560 downregulated), and compounds such as syringaresinol, shilajic acid, and 2-(2-fluorophenyl)-6-methylquinoline-4-carboxamide exhibited marked fluctuations in expression (Figure 7F).

**Figure 8:**
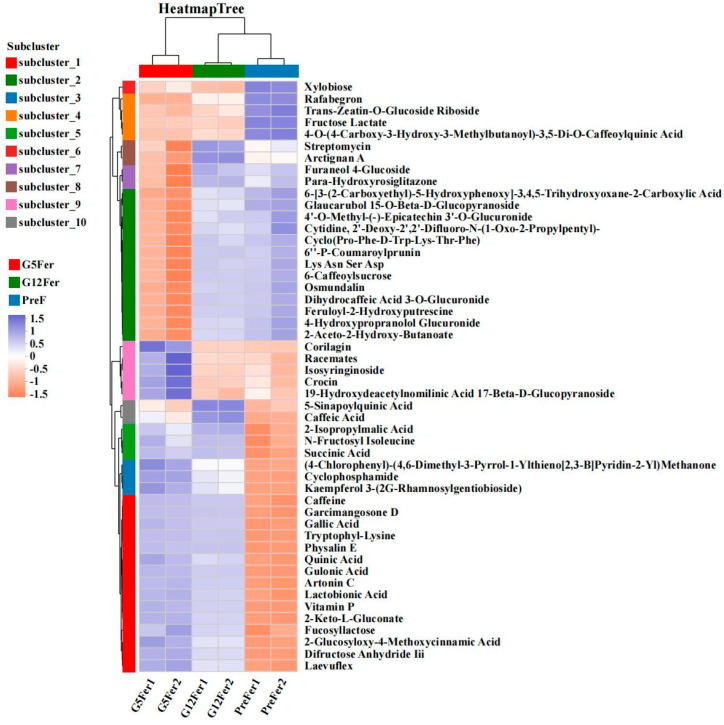
Heatmap analysis of Top 50 significantly differential metabolites (VIP > 1, *p* < 0.05). The bluer the color, the higher the abundance of metabolites, and the oranger the color, the lower the abundance of metabolites.

Based on thresholds of VIP > 1 and *p* < 0.05, the top 50 differential metabolites were screened, identified, and visualized using a clustered heatmap (Figure 8). The heatmap revealed the global similarities and differences in the metabolic profiles of the PreFer, G5Fer, and G12Fer groups. According to the dendrogram clustering structure, samples of the unfermented group (PreFer) and the fermented groups (G5Fer and G12Fer) were clearly separated into two major clusters, indicating that fermentation induced a fundamental remodeling of the metabolic profile of *gardenia* fruit. Notably, characteristic metabolites of kombucha fermentation, such as succinic acid, gulonic acid, 2-keto-L-gluconate, and lactobionic acid, were significantly enriched in the G5Fer and G12Fer groups compared with the PreFer group, reflecting the sustained microbial metabolic activities. Moreover, the levels of polyphenol and phenolic acid compounds such as gallic acid, caffeic acid, quinic acid, corilagin, and vitamin *p* were higher in the G5Fer and G12Fer groups than in the PreFer group, suggesting that fermentation effectively promoted the degradation of complex polyphenols and the release of free phenolic acids. These changes in individual phenolic compounds are consistent with the total polyphenol content results, which showed higher bioactive content in the G5Fer and G12Fer groups (Figure 3). Furthermore, kaempferol-3-O-rutinoside was significantly upregulated in both the G5Fer and G12Fer groups, with fold changes of 10.30 and 9.997, respectively, relative to the PreFer group, indicating that fermentation facilitated the release and accumulation of flavonol glycosides.

Further observation revealed that although the metabolic profiles of the G5Fer and G12Fer groups partially overlapped, they still exhibited a trend of differentiation, suggesting a significant dose-dependent effect of *gardenia* fruit addition on the metabolic profile. Intriguingly, despite this trend of differentiation, a closer inspection of the hierarchical clustering tree (Figure 8) revealed that the global metabolic landscape of the high-dose group (G12Fer) was positioned closer to the unfermented baseline (PreFer) than to the optimized group (G5Fer). This counter-intuitive proximity strongly points to a fermentation-inhibitory effect triggered by excessive substrate addition. *Gardenia* fruit is inherently rich in iridoid glycosides and crocin derivatives, which possess potent, dose-dependent antimicrobial, and membrane-disrupting properties against certain yeasts and acetic acid bacteria. At a 12.5% dosage, the excessive initial concentration of these bio-actives, combined with heightened osmotic pressure, likely suppressed the metabolic vigor or delayed the growth kinetics of the SCOBY consortium. Consequently, both the basal breakdown of tea–sugar constituents and the biotransformation rate of baseline inputs into downstream metabolites were markedly retarded in G12Fer, leaving its global metabolomic landscape closer to its unfermented initial state. Racemates, isosyringinoside, crocin, and 19-hydroxydeacetylnomilinic acid 17-β-D-glucopyranoside were specifically enriched in the G5Fer group, while 5-sinapoylquinic acid and caffeic acid specifically accumulated in the G12Fer group (Figure 8). Notably, the increase in crocin content differed from the trend observed for crocin-I measured by HPLC (Figure 5). This discrepancy may be attributed to the secretion of glycosyltransferases and esterases by yeasts and lactic acid bacteria in the fermentation system, which catalyze the synthesis of crocin precursor (e.g., crocetin) or modify them through glycosylation and esterification, thereby generating crocin derivatives with higher water solubility and stability, such as crocin-3-O-glucoside, crocin-4, and crocin-5 [44].

**Figure 9:**
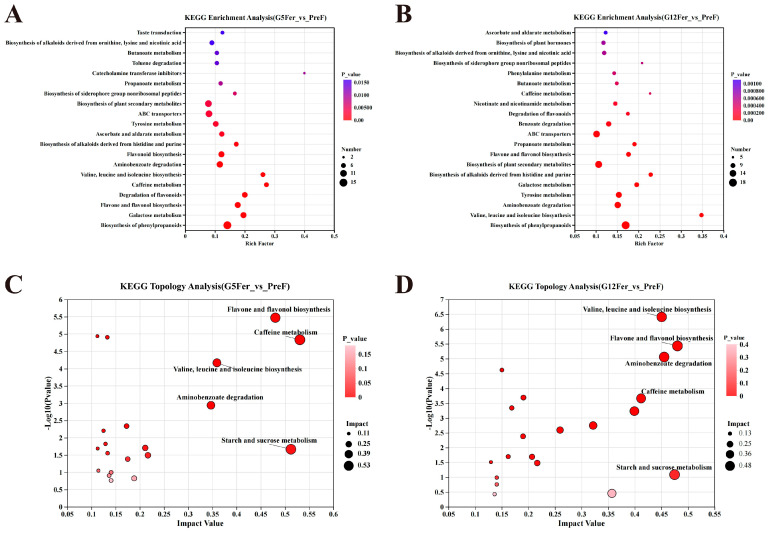
KEGG enrichment of key metabolic pathways. Bubble charts of top 20 metabolic pathway enrichment map between G5Fer group vs. Prefer group (**A**) and G12Fer group vs. Prefer group (**B**). KEGG topology analysis of top 5 metabolic pathways between G5Fer group vs. Prefer group (**C**) and G12Fer group vs. Prefer group (**D**).

Based on the observed changes in compound concentrations, metabolic pathway prediction was conducted using the KEGG database. The results showed that the biosynthesis of phenylpropanoids was the most significantly enriched pathway during the fermentation of *gardenia* fruit in both G5Fer (Figure 9A) and G12Fer groups (Figure 9B). The previous untargeted metabolomic study has confirmed that this pathway is among the most prominently enriched pathways during kombucha fermentation, with downstream flavonoids and phenolic acids accounting for the largest proportion of compounds [48], suggesting that fermentation significantly promotes the biosynthesis and release of active substances such as polyphenols and flavonoid precursors in *gardenia* fruit. Additionally, pathways, including flavone and flavonol biosynthesis, valine, leucine and isoleucine biosynthesis, and caffeine metabolism, were also significantly enriched (Figure 9C,D). In this context, the flavonoid and flavonol biosynthetic pathways are involved in regulating the modification and structural diversification of flavonoid glycosides, ultimately affecting their subsequent metabolic transformation and the accumulation of aglycone flavonoids [7].

### 3.9. Sensory Analysis of Gardenia Fruit Kombucha

Sensory analysis is essential for understanding product acceptance, expectations, emotions, and perceptions, with acceptance testing serving as a key methodology in food science [14]. This study evaluated consumer acceptance of kombucha beverages produced from different amounts of *gardenia* fruit powder infusions on day 8 of fermentation. A panel of 20 trained and experienced members assessed the *gardenia* fruit kombucha samples based on color, aroma, flavor, transparency, and overall acceptance using a nine-point hedonic scale, where higher scores indicated better performance for each attribute.

The data indicate that the amount of *gardenia* fruit added significantly influenced the overall sensory quality of the fermented beverages. Consistent with the appearance observed in Figure 1, the G2.5Fer and G5Fer groups received higher appearance scores, attributable to their appealing color and enhanced transparency. In contrast, for the G7.5Fer, G10Fer, and G12Fer groups, higher addition levels of *gardenia* fruit resulted in lower color and transparency scores due to turbidity and dark coloration (Table 1). Notably, the *gardenia* fruit kombucha samples of the G5Fer group consistently received the highest scores for aroma, flavor, and overall acceptance, indicating greater consumer preference. Specifically, no alcoholic odor was detected throughout the fermentation period, and the sourness was palatable in the G2.5Fer and G5Fer groups. The G7.5Fer group had a slight sour odor, and the G10Fer and G12Fer groups exhibited a strong sour odor by day 8. Concurrently, the gradual increase in sourness perception can be attributed to greater organic acid production at higher *gardenia* fruit addition levels. In contrast, the milder overall sourness in the G5Fer group may result from a decrease in sharp organic acids (e.g., malic and citric acid). Although malic acid offers a green apple-like flavor, it also imparts a metallic taste to fermented products [8]. Meanwhile, although succinic acid—which contributes sour, salty, and bitter notes [8]—increased across all groups, its increase was smallest in the G5Fer group, which also contributes to the improvement in aroma and flavor. Thus, keeping organic acids, which not only contribute to the sour taste but also serve as precursors for various volatile flavor compounds [49], within an appropriate range is more favorable for overall acceptability.

## 4. Conclusions

This study is the first to investigate the use of *gardenia* fruit as a substrate for kombucha production. *Gardenia* fruit powder proved to be a suitable fermentation medium, as demonstrated by variations in key parameters, such as acidity, fermentation metabolites (organic acids, total polyphenol and flavonoid contents, etc.), and sensory attributes. The kombucha fermented with 5.0% *gardenia* fruit powder exhibited thicker SCOBY-cellulose, higher acidity, increased total polyphenols content, and improved sensory characteristics, particularly in aroma, flavor, and overall acceptance. Untargeted metabolomic results further highlight the ability of the kombucha microbial consortium to biotransform *gardenia* fruit compounds into more bioactive forms, such as phenolic acids (e.g., gallic acid, caffeic acid) and flavonol glycosides (e.g., kaempferol-3-O-rutinoside). Moreover, the antioxidant activity of *gardenia* fruit kombucha was maintained after fermentation, reinforcing its potential as functional beverages with health-promoting properties. Overall, this study provides an upcycling approach for developing functional fermented beverages using *gardenia* fruit, which possesses both medicinal and edible properties. This approach aligns with the growing consumer demand for flavored and functional kombucha beverages.

## Figures and Tables

**Table 1:** Sensory acceptance scores for Kombucha produced from *gardenia* fruit.

Sample	Color	Aroma	Flavor	Transparency	Overall Acceptance
G2.5Fer	7.63 ± 0.74 ^a^	7.13 ± 0.83 ^a^	4.50 ± 0.76 ^ab^	4.88 ± 0.83 ^a^	6.75 ± 0.89 ^a^
G5Fer	7.25 ± 0.71 ^a^	7.13 ± 0.99 ^a^	5.50 ± 1.31 ^a^	4.50 ± 0.93 ^ab^	7.75 ± 0.71 ^a^
G7.5Fer	5.75 ± 0.46 ^b^	5.75 ± 1.16 ^b^	3.63 ± 0.92 ^bc^	3.50 ± 0.53 ^bc^	5.25 ± 0.89 ^b^
G10Fer	2.75 ± 0.71 ^c^	2.63 ± 0.52 ^c^	2.88 ± 0.64 ^c^	2.63 ± 0.52 ^cd^	2.88 ± 0.83 ^c^
G12Fer	2.25 ± 0.89 ^c^	2.88 ± 0.64 ^c^	2.75 ± 0.46 ^c^	2.38 ± 0.74 ^d^	2.75 ± 0.71 ^c^

Note: Values are expressed as means ± SD. Different superscript letters within the same column indicate statistically significant differences at *p* < 0.05 (one-way ANOVA followed by Tukey test). G2.5Fer, G5Fer, G7.5Fer, G10Fer, and G12Fer represent fermented *gardenia* fruit kombucha samples collected on Day 8, fortified with 2.5%, 5.0%, 7.5%, 10.0%, and 12.5% (*w*/*v*) of *gardenia* fruit powder, respectively.

## Data Availability

The original contributions presented in this study are included in the article/Supplementary Material. Further inquiries can be directed to the corresponding author.

## Supplementary Materials

The following supporting information can be downloaded at: https://www.mdpi.com/article/10.3390/foods15152670/s1. Supplementary Table S1: The complete dataset and curation metrics of non-targeted metabolomics analysis on *gardenia* fruit kombucha. Supplementary Table S2: The sensory evaluation criteria for the Gardenia Black Tea Beverage. Supplementary Table S3: All annotated compounds of non-targeted metabolomics analysis on *gardenia* fruit kombucha.

## References

[1] Tian Y., Zhou Y., Zhang Y., Ni Q., Yang B. (2026). Compositional diversity of iridoids, phenolic compounds, and crocins in Gardenia jasminoides fruits of different varieties. Food Chem..

[2] Chen L., Li M., Yang Z., Tao W., Wang P., Tian X., Li X., Wang W. (2020). Gardenia jasminoides Ellis: Ethnopharmacology, phytochemistry, and pharmacological and industrial applications of an important traditional Chinese medicine. J. Ethnopharmacol..

[3] Tian J., Qin S., Han J., Meng J., Liang A. (2022). A review of the ethnopharmacology, phytochemistry, pharmacology and toxicology of Fructus Gardeniae (Zhi-zi). J. Ethnopharmacol..

[4] Jin C., Zongo A.W., Du H., Lu Y., Yu N., Nie X., Ma A., Ye Q., Xiao H., Meng X. (2025). Gardenia (*Gardenia jasminoides* Ellis) fruit: A critical review of its functional nutrients, processing methods, health-promoting effects, comprehensive application and future tendencies. Crit. Rev. Food Sci. Nutr..

[5] Liu L., Wu Q., Chen Y., Gu G., Gao R., Peng B., Wang Y., Li A., Guo J., Xu X. (2022). Updated pharmacological effects, molecular mechanisms, and therapeutic potential of natural product geniposide. Molecules.

[6] Lu X., Li J., Ma Y., Khan I., Yang Y., Li Y., Wang Y., Liu G., Zhang Z., Yang P. (2023). Fermented Angelica sinensis activates Nrf2 signaling and modulates the gut microbiota composition and metabolism to attenuate D-gal induced liver aging. Food Funct..

[7] Wei Y., Zhang G., Fei T., Lin X., Wang L. (2026). Unveiling the formation mechanism of Moringa oleifera leaf flavonoids aglycones during Monascus anka fermentation by using integrated widely targeted metabolomics and proteomics analysis. Food Chem..

[8] Wang L., Wang E., Wang W., Zhang W., Ge Y., Cao X., Tian H.S., Li X., Zhu X. (2026). Eucommia ulmoides leaf as an alternative substrate for kombucha fermentation: The role of microbial communities in dynamics and metabolic profile. Food Chem..

[9] Nunes G., Sola I.M.M.S., Fischer T.E., Nogueira A., Alberti A. (2025). Impact of Handroanthus albus extract on the bioactive composition and fermentation kinetics of kombucha. Food Chem..

[10] Li S., Chu K., Guo R., Wu J., Wang X., Guo J., Yuan Y., Yue T., Cai R., Wang Z. (2025). Tailoring a simplified and steady-state kombucha consortium for newly-formulated kiwifruit beverage production: Impacts on untargeted/targeted metabolites, antioxidant capacities and flavor profiles. Food Res. Int..

[11] de Lima A.S.L., de Medeiros Felipe A.T., de Oliveira Paiva E.M., Medeiros R.D., de Sousa Junior F.C., Matsui K.N., Zucolotto S.M., da Silva Pedrini M.R. (2025). Fermentation of passion fruit leaf tea with Kombucha inoculum: An upcycling approach for the development of functional fermented beverages. Food Res. Int..

[12] Barros V.C., Botelho V.A., Chisté R.C. (2024). Alternative substrates for the development of fermented beverages analogous to kombucha: An integrative review. Foods.

[13] Wu S.X., Xiong R.G., Cheng J., Xu X.Y., Tang G.Y., Huang S.Y., Zhou D.D., Saimaiti A., Gan R.Y., Li H.B. (2023). Preparation, antioxidant activities and bioactive components of kombucha beverages from golden-flower tea (*Camellia petelotii*) and honeysuckle-flower tea (*Lonicera japonica*). Foods.

[14] Bressani A.P.P., Casimiro L.K.S., Martinez S.J., Dias D.R., Schwan R.F. (2024). Kombucha with yam: Comprehensive biochemical, microbiological, and sensory characteristics. Food Res. Int..

[15] Aung T., Eun J.B. (2021). Production and characterization of a novel beverage from laver (*Porphyra dentata*) through fermentation with kombucha consortium. Food Chem..

[16] Tong K., Liao Y., Tang Y., Luo Y., Liu X., Yu D., Zhou J., Hou C., Li Z. (2026). Red Yeast Rice-Driven Kombucha Fermentation: A Novel Strategy for Developing Functional Beverages with Enhanced Hypoglycemic and Hypolipidemic Properties. Foods.

[17] Sánchez-Rangel J.C., Benavides J., Heredia J.B., Cisneros-Zevallos L., Jacobo-Velázquez D.A. (2013). The Folin–Ciocalteu assay revisited: Improvement of its specificity for total phenolic content determination. Anal. Methods.

[18] (2023). Sensory Analysis—Selection and Training of Sensory Assessors.

[19] Chandran A., Wyka J., Klein G.-R., Stefanska B., Kolniak-Ostek J. (2026). Shaping the Bioactive Properties of Kombucha Drinks by Using Raw Materials Alternative to Tea. Molecules.

[20] da Silva S.F., Cavalcante M.P., Sensheng Y., Silva S.d.S., Frota Gaban S.V. (2024). Physicochemical properties, antioxidant activity, and sensory profiles of kombucha and kombucha-like beverages prepared using passion fruit (*Passiflora edulis*) and apple (*Malus pumila*). ACS Agric. Sci. Technol..

[21] de Oliveira Duarte F.A., Ramos K.K., Gini C., Morasi R.M., Silva N.C.C., Efraim P. (2024). Microbiological characterization of kombucha and biocellulose film produced with black tea and cocoa bean shell infusion. Food Res. Int..

[22] Villarreal-Soto S.A., Beaufort S., Bouajila J., Souchard J.P., Taillandier P. (2018). Understanding kombucha tea fermentation: A review. J. Food Sci..

[23] Chen A., Li J., Yao A., Du G., Li J., Chen J. (2025). Advancing kombucha fermentation: Microbial interactions, functional metabolites, and innovative optimization strategies. Food Chem..

[24] Wang S., Li C.W., Wang Y., Wang S., Zou Y.P., Sun Z., Yuan L. (2023). Changes on physiochemical properties and volatile compounds of Chinese kombucha during fermentation. Food Biosci..

[25] Su J., Tan Q., Tang Q., Tong Z., Yang M. (2023). Research progress on alternative kombucha substrate transformation and the resulting active components. Front. Microbiol..

[26] Saichana N., Matsushita K., Adachi O., Frébort I., Frebortova J. (2015). Acetic acid bacteria: A group of bacteria with versatile biotechnological applications. Biotechnol. Adv..

[27] Wang Y., Wu J., Lv M., Shao Z., Hungwe M., Wang J., Bai X., Xie J., Wang Y., Geng W. (2021). Metabolism characteristics of lactic acid bacteria and the expanding applications in food industry. Front. Bioeng. Biotechnol..

[28] Xing Z., Belwal T., Huang H., Li X., Han L., Yin Y., Shan C., Fu X., Du Y. (2025). Combining untargeted metabolomics and chemometrics to reveal the metabolic profiles of jujube-hawthorn fermented beverages at different fermentation stages. Food Chem..

[29] Tran T., Grandvalet C., Verdier F., Martin A., Alexandre H., Tourdot-Maréchal R. (2020). Microbial dynamics between yeasts and acetic acid bacteria in kombucha: Impacts on the chemical composition of the beverage. Foods.

[30] Li K., Yu L., Gao L., Zhu L., Feng X., Deng S. (2024). Unveiling molecular mechanisms of pigment synthesis in gardenia (*Gardenia jasminoides*) fruits through integrative transcriptomics and metabolomics analysis. Food Chem. Mol. Sci..

[31] Xiao W., Li S., Wang S., Ho C.T. (2017). Chemistry and bioactivity of *Gardenia jasminoides*. J. Food Drug Anal..

[32] Bangar S.P., Suri S., Trif M., Ozogul F. (2022). Organic acids production from lactic acid bacteria: A preservation approach. Food Biosci..

[33] Yang B., Li Q., Cheng K., Fang J., Mustafa G., Pan J., Xing B., Lv Q., Zhang L., Cheng K. (2022). Proteomics and metabolomics reveal the mechanism underlying differential antioxidant activity among the organs of two base plants of Shiliang tea (*Chimonanthus salicifolius* and *Chimonanthus zhejiangensis*). Food Chem..

[34] Rahmani R., Beaufort S., Villarreal-Soto S.A., Taillandier P., Bouajila J., Debouba M. (2019). Kombucha fermentation of African mustard (*Brassica tournefortii*) leaves: Chemical composition and bioactivity. Food Biosci..

[35] Zhu K., Ouyang J., Huang J., Liu Z. (2021). Research progress of black tea thearubigins: A review. Crit. Rev. Food Sci. Nutr..

[36] Wang X., Wang D., Wang H., Jiao S., Wu J., Hou Y., Sun J., Yuan J. (2022). Chemical profile and antioxidant capacity of kombucha tea by the pure cultured kombucha. LWT.

[37] Tu C.H., Yu T.F., Feng S.B., Xu N., Massawe A., Shui S.S., Zhang B. (2024). Dynamics of microbial communities, flavor, and physicochemical properties of kombucha-fermented Sargassum fusiforme beverage during fermentation. LWT-Food Sci. Technol..

[38] Zofia N., Aleksandra Z., Tomasz B., Martyna Z.D., Magdalena Z., Zofia H.B., Tomasz W. (2020). Effect of fermentation time on antioxidant and anti-ageing properties of green coffee kombucha ferments. Molecules.

[39] Reddy Y.M., Kumar S.P.J., Saritha K.V., Gopal P., Reddy T.M., Simal-Gandara J. (2021). Phytochemical Profiling of Methanolic Fruit Extract of *Gardenia latifolia* Ait. by LC-MS/MS Analysis and Evaluation of Its Antioxidant and Antimicrobial Activity. Plants.

[40] Liang W., Wang X., Zhang L., Jiao S., Song H., Sun J., Wang D. (2024). Changes and biotransformation mechanism of main functional compounds during kombucha fermentation by the pure cultured tea fungus. Food Chem..

[41] Wei Y., Chen Y., Lin X., Zhang S., Zhu B., Ji C. (2025). Integrated transcriptome and proteome analysis unveils black tea polyphenols metabolic pathways in Saccharomyces cerevisiae. Food Microbiol..

[42] Amarasinghe H., Weerakkody N.S., Waisundara V.Y. (2018). Evaluation of physicochemical properties and antioxidant activities of kombucha “Tea Fungus” during extended periods of fermentation. Food Sci. Nutr..

[43] Yang S., Liu Y., Tan W., Ren J., Hou M., Wang Z., Guo C., Gao Z. (2025). Lactic acid bacteria fermentation of prebiotic-supplemented apple juice: Viable counts and flavor evolution revealed by HS-SPME-GC–MS coupled with electronic sensory and chemometrics. Food Chem..

[44] Wang X., Zhuhuang C., He Y., Zhang X., Wang Y., Ni Q., Zhang Y., Xu G. (2024). Selective transformation of crocin-1 to crocetin-glucosyl esters by β-glucosidase (Lf18920) from *Leifsonia* sp. ZF2019: Insights from molecular docking and point mutations. Enzym. Microb. Technol..

[45] Cui R., Zhang C., Pan Z.H., Hu T.G., Wu H. (2024). Probiotic-fermented edible herbs as functional foods: A review of current status, challenges, and strategies. Compr. Rev. Food Sci. Food Saf..

[46] Uddin R., Saha M.R., Subhan N., Hossain H., Jahan I.A., Akter R., Alam A. (2014). HPLC-analysis of polyphenolic compounds in *Gardenia jasminoides* and determination of antioxidant activity by using free radical scavenging assays. Adv. Pharm. Bull..

[47] Peng X., Yang S., Liu Y., Ren K., Tian T., Tong X., Dai S., Lyu B., Yu A., Wang H. (2023). Application of kombucha combined with fructo-oligosaccharides in soy milk: Colony composition, antioxidant capacity, and flavor relationship. Food Biosci..

[48] Li S., Wang S., Wang L., Liu X., Wang X., Cai R., Yuan Y., Yue T., Wang Z. (2023). Unraveling symbiotic microbial communities, metabolomics and volatilomics profiles of kombucha from diverse regions in China. Food Res. Int..

[49] Yoon K.N., Song S.H., Jang D., Kim M.H., Hong J.W., Won Y.S., Lee S.J., Min A.M.J., Kim Y.M., Cho J.Y. (2026). Application of kombucha fermentation to improve the stability of bioactive metabolites in purple corn (*Zea mays* L.) cob extract. LWT-Food Sci. Technol..

